# Rapid whole exome sequencing in pregnancies to identify the underlying genetic cause in fetuses with congenital anomalies detected by ultrasound imaging

**DOI:** 10.1002/pd.5717

**Published:** 2020-05-05

**Authors:** Chantal Deden, Kornelia Neveling, Dimitra Zafeiropopoulou, Christian Gilissen, Rolph Pfundt, Tuula Rinne, Nicole de Leeuw, Brigitte Faas, Thatjana Gardeitchik, Suzanne C. E. H. Sallevelt, Aimee Paulussen, Servi J. C. Stevens, Esther Sikkel, Mariet W. Elting, Merel C. van Maarle, Karin E. M. Diderich, Nicole Corsten‐Janssen, Klaske D. Lichtenbelt, Guus Lachmeijer, Lisenka E. L. M. Vissers, Helger G. Yntema, Marcel Nelen, Ilse Feenstra, Wendy A. G. van Zelst‐Stams

**Affiliations:** ^1^ Department of Human Genetics Radboud University Medical Center, Radboud Institute for Health Sciences Nijmegen The Netherlands; ^2^ Department of Genetics, University Medical Center Groningen University of Groningen Groningen The Netherlands; ^3^ Department of Human Genetics Radboud University Medical Center, Radboud Institute for Molecular Life Sciences Nijmegen The Netherlands; ^4^ Department of Human Genetics, Donders Institute for Brain, Cognition, and Behaviour Radboud University Medical Center Nijmegen Netherlands; ^5^ Department of Clinical Genetics Maastricht University Medical Center Maastricht The Netherlands; ^6^ Department of Obstetrics and Gynecology Radboud University Medical Centre Nijmegen The Netherlands; ^7^ Department of Clinical Genetics AMsterdam UMC, Vrije Universiteit Amsterdam Amsterdam The Netherlands; ^8^ Department of Clinical Genetics AMsterdam UMC, University of Amsterdam Amsterdam The Netherlands; ^9^ Department of Clinical Genetics Erasmus University Medical Center Rotterdam Rotterdam The Netherlands; ^10^ Department of Genetics Utrecht University Medical Center Utrecht The Netherlands

## Abstract

**Objective:**

The purpose of this study was to explore the diagnostic yield and clinical utility of trio‐based rapid whole exome sequencing (rWES) in pregnancies of fetuses with a wide range of congenital anomalies detected by ultrasound imaging.

**Methods:**

In this observational study, we analyzed the first 54 cases referred to our laboratory for prenatal rWES to support clinical decision making, after the sonographic detection of fetal congenital anomalies. The most common identified congenital anomalies were skeletal dysplasia (n = 20), multiple major fetal congenital anomalies (n = 17) and intracerebral structural anomalies (n = 7).

**Results:**

A conclusive diagnosis was identified in 18 of the 54 cases (33%). Pathogenic variants were detected most often in fetuses with skeletal dysplasia (n = 11) followed by fetuses with multiple major fetal congenital anomalies (n = 4) and intracerebral structural anomalies (n = 3). A survey, completed by the physicians for 37 of 54 cases, indicated that the rWES results impacted clinical decision making in 68% of cases.

**Conclusions:**

These results suggest that rWES improves prenatal diagnosis of fetuses with congenital anomalies, and has an important impact on prenatal and peripartum parental and clinical decision making.

**What's already known about this topic?**

- Several pilot studies report on an added value of prenatal rapid whole exome sequencing (rWES), when routine techniques fail to identify a genetic diagnosis in fetuses with congenital anomalies detected by ultrasound imaging.

**What does this study add?**

- We determined the diagnostic yield for rWES in 54 cases with fetal congenital anomalies detected by ultrasound imaging in pregnancies being 33% and show its impact on clinical decision making.
- Rapid aggregation of prenatal molecular and clinical information into a conclusive diagnosis is challenging and requires cooperation of a dedicated team.

## 1 INTRODUCTION

Fetal congenital anomalies are detected in 2% to 5% of pregnancies by routine ultrasound.^1^, ^2^ The occurrence of these anomalies can cause significant distress for the expecting parents and have a major impact on perinatal mortality and long‐term morbidity.^3^, ^4^ The underlying etiology of these anomalies is diverse and includes genetic factors. Current routine prenatal genetic testing strategies often include molecular rapid aneuploidy testing (RAD) and chromosomal microarray analysis (CMA), designed to detect numerical and structural chromosome abnormalities, respectively, which show a combined diagnostic yield of approximately 40%.^5^, ^6^ However, this means that for the large majority of cases, the underlying cause of the identified congenital anomalies remains unknown. The latter is most prominent for congenital anomalies that are a result of monogenic disorders caused by point mutations and/or small insertion deletion events. Whole exome sequencing (WES) in a postnatal setting has shown to increase diagnostic yield for genetically heterogeneous (monogenic) disorders to up to 58%, depending on the clinical preselection of the cohort and subset(s) of genes analyzed.^7^, ^8^, ^9^ The turn‐around times (TATs) of routine WES, being several months, has so far always hampered this assay to be implemented in routine prenatal diagnostics. A decrease of this TAT may help to diagnose those fetuses with congenital anomalies in which the genetic diagnosis remained elusive using routine prenatal procedures.

Rapid whole exome sequencing (rWES), with TATs varying from 4 days to several weeks, has been shown to contribute to clinical decision making in pediatric and neonatal critical care.^10^, ^11^, ^12^, ^13^ It is very likely that rWES has the same potential for prenatal clinical decision making. In a recent study on the use of rWES for fetuses presenting with skeletal anomalies, 81% of cases were genetically diagnosed.^14^ Although this increase in diagnoses enabled more accurate prediction of pregnancy outcome, providing parents more certainty in prenatal decision making, the contribution of skeletal anomalies only accounts for around 30% of all fetal congenital anomalies.^15^, ^16^ The efficacy of adopting rWES as a first tier test for the full spectrum of fetal congenital anomalies detected during routine ultrasound imaging has also been recently studied in a few pilot studies.^17^, ^18^, ^19^, ^20^ The vast majority of these studies focused on the diagnostic yield and TAT as outcome parameters, rather than focusing the effect of the rWES result on clinical decision making. Here we report the rWES results of 54 fetuses with congenital anomalies in ongoing pregnancies and highlight the effect of rWES on clinical decision making.

## 2 METHODS

### 2.1 Patient eligibility and selection for prenatal rWES

Since January 2016, rWES has been offered as a routine diagnostic test at the Radboudumc for cases whose medical management could be directly impacted by a genetic diagnosis. For prenatal cases, rWES was offered following the detection of multiple fetal congenital anomalies suggestive of a possible genetic etiology detected by ultrasound imaging in level III academic centers, executed or supervised by Maternal Fetal Medicine specialists. In case of an isolated major anomaly or (multiple) soft markers,^21^ rWES was only offered if there was a high suspicion of a genetic cause. A detailed case by case description of the clinical presentation is provided in Appendix S1. Fetal materials derived from a pregnancy that had ended in fetal death, or from a termination of pregnancy (TOP) were not included in this study.

### 2.2 Informed consent and counseling

Patients received pre‐ and posttest rWES counseling by a clinical geneticist. Diagnostic informed consent was identical to our routine postnatal procedure, and comprised of a two‐tiered process to limit the chance of uncovering incidental findings. In tier 1, interpretation is focused toward gene variants in dedicated (in silico) disease gene panel(s), which are selected by the clinical geneticist based on the clinical presentation of the fetus. In this study, 12 different in silico disease‐gene panels were used, in size ranging between 48 genes for “Fetal Akinesia” and 1158 for “Intellectual Disability” (Table 1, Appendix S1). To allow immediate interpretation of all genes with a known disease phenotype in tier 1, “The Mendeliome Panel” (also referred to as clinical exome) can be requested, containing 3605 genes with well‐established genotype–phenotype associations. In tier 2, generally only performed after a negative (or possible) result for the in silico disease gene panel(s), interpretation is extended to the Mendeliome as well as all other protein‐coding genes with currently unknown disease‐relationships. In this study, tier 2 analysis was performed for 12 cases, of whom eight cases already had had a negative Mendeliome analysis in tier 1 (Appendix S1). An overview of all genes included in the in silico disease‐gene panels, as well as our policy for disclosing of incidental findings, can be found online in https://order.radboudumc.nl/en/genetics/rapid-exome-sequencing.

**TABLE 1:** Overview of cohort characteristics and rWES analysis and interpretation strategies

Cohort characteristics	Number
**Number of cases, n**	54
**Referring academic medical center, n (%)**	
Amsterdam University Medical Centre (AUMC)	5 (9%)
Erasmus Medical Centre (EMC)	4 (7%)
Maastricht University Medical Centre (MUMC+)	10 (19%)
Radboud University Medical Centre (RUMC)	18 (33%)
University Medical Centre Groningen (UMCG)	1 (2%)
University Medical Centre Utrecht (UMCU)	16 (30%)
**Pregnancy duration, median in weeks (range)**	21w5d (17w5d‐39w1d)
**Maternal age, median in years (range)**	30 (20‐39)
**Primary clinical feature**	
Skeletal dysplasia	20 (37%)
MFCA^a^	17 (31%)
Intracerebral structural anomalies	7 (13%)
Other^b^	10 (19%)
**Chromosomal Microarray analysis ** **(CMA)**	
Normal CMA result prior to rWES	22 (41%)
CMA and rWES in parallel	25 (46%)
No CMA performed	2 (4%)
Unknown	5 (9%)

rWES analysis and Interpretation strategies^c^	Number
**rWES analytical approach**	
Trio‐based analysis	53 (98%)
**Clinical exome interpretation**	32 (59%)
Mendeliome	25
Mendeliome +1 other in silico disease gene panel	4
*+ Intellectual disability*	1
*+ Congenital heart disease*	1
*+ Craniofacial anomalies*	1
*+ Skeletal dysplasia and short stature*	1
Mendeliolome +2 other in silico disease gene panels	1
*+ Skeletal dysplasia and short stature + metabolic disorders*	1
Mendeliolome +3 other in silico disease gene panels	2
*+ Intellectual disability + Movement disorders + Epilepsy*	1
*+ Skeletal dysplasia and short stature + disorders of sexual development + vision disorders*	1
**Initial interpretation only for in silico disease‐gene panel (s)**	22 (41%)
1 in silico disease gene panel	21
*Skeletal dysplasia and short stature*	20
*Disorders of sex development*	1
3 in silico disease gene panels	1
*Fetal Akinesia + muscle disorders + hereditary motor sensory neuropathy*	1

a MFCA: multiple fetal congenital anomalies.

b Other: anomalies such as congenital diaphragmatic hernia or fetal akinesia.

c Analytical details per case are listed in Appendix S1.

Abbreviation: rWES, rapid whole exome sequencing.

### 2.3 rWES

For fetal samples, DNA was isolated from fetal material without culturing of cells, whereas DNA from the parental samples was isolated from blood obtained through venipuncture. DNA library preparation was performed using SureSelect QXT in combination with the Sure Select All Human Exon Kit (v5, Agilent), followed by 2x150bp paired‐end sequencing on a NextSeq500 (Illumina). Sequence coverage was 200 to 300×. Automated data analysis pipeline included rapid BWA mapping, GATK variant calling and custom‐made annotation. Parental and fetal DNA was sequenced simultaneously in 53 of 54 cases (trio‐based analysis) to favor interpretation of results. For the remaining case, paternal DNA was unavailable.

Prior to rWES, aneuploidies for trisomy 13, 18 and 21 and monosomy X were excluded in all cases by quantitative fluorescent polymerase chain reaction (QF‐PCR) using routine procedures.^22^ Additionally, CMA was performed prior to (n = 22, 41%), or in‐parallel with (n = 25, 46%) rWES.

### 2.4 Variant classification

Classification of variant pathogenicity for single nucleotide variants (SNVs) or copy number variations (CNVs) was based on European guidelines.^23^, ^24^ If insufficient clinical information, or potential discrepancy between molecular and clinical causality was noted, variants were discussed in a multidisciplinary team, consisting of a clinical laboratory geneticist, a clinical geneticist and a fetal maternal specialist. Overall, this resulted in reporting of (likely) pathogenic variants (class 4 and 5) related to the fetal phenotype, as well as the reporting of variants of unknown significance (VUS; class 3) if the multidisciplinary team concluded that the VUS was likely to contribute to the fetal phenotype.

### 2.5 Primary end points

Primary end points were (a) diagnostic yield, (b) TAT (in calendar days) and (c) the influence of rWES in perinatal clinical decision making. The diagnostic yield was defined as the percentage of cases from the total cohort for whom the genetic variant (likely) explained the identified phenotype. The TAT was measured from the moment the rWES was requested until the return of the written diagnostic rWES report. The influence of rWES in perinatal clinical decision making was analyzed with the help of a survey sent to the requesting clinicians to collect additional data related to, amongst others, the reason for the rWES request, the pregnancy outcome, and management adaptations (Appendix S1). Impact was defined as to influence the decision:

- To opt for a TOP before 24 weeks of gestation, which—in the Netherlands—is the legal limit for a TOP; and/or
- To request for a late TOP after 24 weeks of pregnancy, which—in the Netherlands—is only allowed when a severe fetal outcome is imminent; and/or
- To continue the pregnancy; and/or
- To adjust peripartum management.

To assess the representativeness of the responses for the total cohort, we compared the diagnostic yield in responders and non‐responders using a Fisher's exact test.

## 3 RESULTS

### 3.1 Cohort characteristics

From May 2016 to November 2018, we received 54 requests for prenatal rWES after the identification of fetal congenital anomalies. The median gestational age was 21 weeks and 5 days (range: 16 + 5 to 38 + 1 weeks) and the median maternal age was 30 years (range: 20‐38 years).

The most common clinical indications were skeletal dysplasia (n *=* 20; 37%), multiple major fetal congenital anomalies (n *=* 17; 31%) and intracerebral structural anomalies (n *=* 7; 13%). Ten cases presented with other congenital anomalies, such as congenital diaphragmatic hernia, fetal akinesia and ambiguous genitalia. An overview of the cohort characteristics is provided in Table 1, with details in Supplementary Table 1.

### 3.2 Clinically relevant CMA results prior to, or in parallel with, rWES

A (likely) pathogenic CNV was detected in two of the 47 cases for whom CMA was performed prior to, or in parallel with, rWES: in case #51 a de novo pathogenic deletion of 17p13.3 (Miller‐Dieker Lissencephaly syndrome, OMIM #247200) was detected in the medical center of referral (Appendix S1). This copy number variant, although retrospectively identified in rWES, was initially not reported by rWES, as analysis was restricted to the gene panel requested which did not include the genomic loci of the genes in the 17p13.3 region. In case #54, CMA detected a potential clinically relevant paternal microdeletion of 1q21.1 including *RBM8A*. Simultaneous analysis of the rWES detected a maternal pathogenic *RBM8A* SNV as well as the paternal 1q21.1 microdeletion, together explaining the phenotype of thrombocytopenia absent radius syndrome (TAR syndrome, OMIM #27400) observed in the fetus.

### 3.3 rWES procedure and turn‐around time

rWES interpretation was guided by in silico analysis of gene panels as requested by the referring physician and to reflect the clinical features observed on ultrasound (Appendix S1). The most commonly requested were the panels “Mendelian inherited disorders” (59%, n = 32/54) and “short stature/skeletal dysplasia” (43%, n = 23/54). A combination of multiple panels was requested for eight patients. For 16 of 54 patients (30%), exome wide analysis (tier 2) was requested in case panel analysis (tier 1) would not result in a diagnosis. In four of these 16, a causative variant was identified within the gene panel, and therefore exome wide analysis was only performed for the remaining 12 patients. The median TAT for rWES was 10 days (range 4‐28 days) and was lowest in case #22 (4 days) since DNA isolation had already been performed at the referring medical center at the time of rWES request. Cases whose analysis was restricted to panel analysis (n *=* 42) had an identical median TAT (10 days) as those for whom exome wide analysis was also performed (n *=* 12).

### 3.4 Diagnostic yield of rWES

**FIGURE 1:**
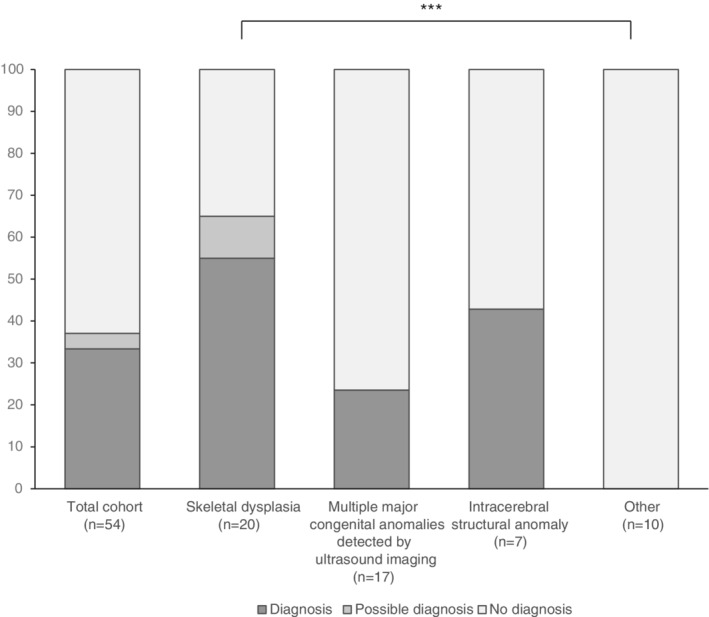
**Overview of total diagnostic yield and per indication of identified fetal congenital anomalies**. Graphical representation of the overall diagnostic yield of prenatal rapid whole exome sequencing. In addition, the yields of each clinical indication are provided. Diagnostic yields were compared to one another to assess whether diagnostic yields differed by clinical cohort. Only statistical significant comparisons are indicated (*: *P* < .05; **: *P* < .01; ***: *P* < .005), highlighting that overall, the fetuses with “other” clinical features than skeletal dysplasia, multiple fetal ultrasound anomalies or intracerebral structural anomalies have a reduced chance on a diagnosis

A conclusive diagnosis was obtained in 18 of the 54 cases (33%) (Figure 1; Table 2; Appendix S1). The highest diagnostic yield was obtained for skeletal dysplasia (61%, n = 11/18), followed by multiple major fetal congenital anomalies (22%, n = 4/18) and intracerebral structural anomalies (17%, n = 3/18; Figure 1). In the ten cases with other congenital anomalies, no molecular diagnoses were made (Figure 1). Among the 18 diagnoses, autosomal dominant (AD) disorders accounted for 72% (n = 13), of which the majority was caused by a de novo variant (n *=* 11/13; 85%). Autosomal recessive (AR) disorders were diagnosed in the remaining five (28%). Interestingly, in case #7, a homozygous pathogenic variant was detected in *ERCC5*, which had resulted from maternal segmental uniparental isodisomy of the distal part of chromosome 13q. The isodisomic segment was confirmed after re‐analysis of the CMA data, which was performed prior to the rWES and was reported as normal.

**TABLE 2:** Overview of diagnoses identified prenatally with the use of rWES

Case description			Clinically relevant variants identified by rWES									Interpretation based on follow up		T
Case ID	Clinical details¥	Indication	Gene	Variant(s)	Protein effect(s)	Zygosity and inheritance		Variant classification based on ACMG guidelines^24^		Phenotype explained	rWES interpretation strategy that identified the pathogenic variant(s)	Follow‐up information	Phenotype explained	Disease (#OMIM; Inheritance pattern)
7	IUGR, short long bones (P0‐P1). Ear anomalies. Clenched hands. Rocker bottom feet. Kidney defect	MFCA	*ERCC5*	NM_000123.3: c.1096C > T	p.(Arg366*)	Hom	Mat	P	PVS1, PM2, PM3	Yes	Clinical Exome (tier 1)	n.a.	n.a.	COFS syndrome type 3 (#616570; AR)
8	Possible skeletal dysplasia. All long bones <P3, OFC P90	SD	*COL2A1*	NM_001844.4: c.1115G > A	p.(Gly372Glu)	Het	UK	LP	PM2,PM5, PP3, PP5	Yes	In silico disease‐gene panel (tier 1)	n.a.	n.a.	Spondyloperipheral dysplasia with short ulna (#27100; AD)
10	Long bones <P3, sandal gap feet. Slight bowing in humeri and femora. Narrow thorax	SD	*FGFR3*	NM_000142.4: c.742C > T	p.(Arg248Cys)	Het	DN	P	PS1, PM1,PM2,PP3, PP5	Yes	In silico disease‐gene panel (tier 1)	n.a.	n.a.	Thanatophoric dysplasia (#187600; AD) or Achondroplasia (#100800; AD)
11	Unilateral bowed and short femur P10	SD	*COL1A2*	NM_000089.3: c.1009G > A	p.(Gly337Ser)	Het	Mat mosaic (27% of reads)	P	PS1, PM1, PM2, PP3, PP5	Yes	In silico disease‐gene panel (tier 1)	n.a.	n.a.	Osteogenesis Imperfecta (Type 2, 3 or 4) (#166210; 259 420; 166 220; AD)
12	CDH, polyhydramnion, hydrothorax and ascites	MFCA	*ANKRD11*	NM_001256182.1: c.6504del	p.(Ala2170fs)	Het	DN	P	PVS1, PS2	Yes	Clinical Exome (tier 1)	n.a.	n.a.	KBG syndrome§ (#148050; AD)
13	Severe skeletal dysplasia	SD	*SLC26A2*	NM_000112.3: c.532C > T NM_000112.3: c.835C > T	p.(Arg178*) p.(Arg279Trp)	CH	Pat Mat	P LP	PVS1, PS3, PM2 PM3, PP5 PS1, PM3, PP3, PP5	Yes	In silico disease‐gene panel (tier 1)	n.a.	n.a.	Diastrophic dysplasia (#222600; AR)
14	Severe skeletal dysplasia: narrow and short thorax. Absence of ossification of sacrum. Brachycephaly.	SD	*COL2A1*	NM_001844.4: c.1879G > C	p.(Gly627Arg)	Het	DN	LP	PS2, PM2, PP3	Yes	In silico disease‐gene panel (tier 1)	n.a.	n.a.	Hypochrondogenesis (#200610; AD)
15	Turribrachycephaly, proptosis, lobar holoprosencephaly, vertebral anomalies	MFCA	*FGFR2*	NM_000141.4: c.1052C > G	p.(Ser351Cys)	Het	DN	P	PS1, PS2, PM2, PP3 PP4	Yes	In silico disease‐gene panel (tier 1)	n.a.	n.a.	Pfeiffer syndrome Antley‐Bixler syndrome (#101600; 207 410; AD)
16	Short long bones, bowed femur, clubfeet, deviation of hand, scoliosis, micrognathia, abnormal filling stomach	SD	*NEK9*	NM_033116.5: c.1871A > G NM_033116.5: c.329_331del	p.(Asn624Ser) p.(Asn110del)	CH	Mat Pat	VUS VUS	PM2, PP3 PM2, PP3	Unclear	In silico disease‐gene panel (tier 1)	Development of arthrogryposis in current pregnancy and in new pregnancy of fetus that also carried both *NEK9* variants.	Yes	Arthrogryposis, Perthes disease, and upward gaze palsy (#614262; AR)
17	Progressive shortening of long bones, deviation of left foot, polyhydramnion.	SD	*COL2A1*	NM_001844.4: c.905C > T	p.(Ala302Val)	Het	DN	P	PS1, PS2, PM2, PM2, PP3, PP5	Yes	In silico disease‐gene panel (tier 1)	n.a.	n.a.	Kniest dysplasia (#156550; AD)
25	Short limbs, short ribs, hypertelorism, skull dysmorphism	SD	*RUNX2*	NM_001024630.3: c.625C > T	p.(Gln209*)	Het	DN	P	PVS1, PS2, PM2, PP5	Yes	In silico disease‐gene panel (tier 1)	n.a.	n.a.	Cleidocranial dysplasia (#119600; AD)
26	Short limbs (femur P0/P0,1), small stomach, plump hands, polyhydramnion.	SD	*COL2A1*	NM_001844.4: c.1286G > A	p.(Gly429Asp)	Het	DN	LP	PS2, PM2, PP3, PP5	Yes	In silico disease‐gene panel (tier 1)	n.a.	n.a.	Type II collagen disorders (range from mild to lethal skeletal dysplasia) (#120140; AD)
30	Microcephaly, abnormal development of sulci, possible neural migration disorder.	ICSA	*TUBA1A*	NM_001270399.1: c.1205G > A	p.(Arg402His)	Het	DN	P	PS2,PM1, PM2, PP3, PP5	Yes	Clinical Exome (tier 1)	n.a.	n.a.	Lissencephaly type 3 (#611603; AD)
33	Hydrocephalus, dysplastic fourth ventricle. Vermis hypoplasia. Lissencephaly. Fold in brainstem.	ICSA	*FKRP*	NM_001039885.2: c.1181G > T	p.(Trp394Leu)	Hom	Pat + Mat	VUS	PM2, PP3, PP4	Yes	Clinical Exome (tier 1)	n.a.	n.a.	FRKP‐related Walker‐Warburg syndrome or Muscle‐Eye‐Brain disease (#613153; AR)
39	Mild bowing of femoral bones with normal length.	SD	*DYNC2H1*	NM_001080463.1: c.1151C > T NM_001080463.1: c.11488_11489del	p.(Ala384Val) p.(Gln3830fs)	CH	Pat Mat	P P	PS1, PM1, PM2, PM3, PP3, PP5 PVS1, PM2, PM3	Yes	In silico disease‐gene panel (tier 1)	n.a.	n.a.	Short rib thoracic dysplasia type 3 with or without polydactyly (#613091; AR)
40	Mild ventriculomegaly, cerebellar hypoplasia, ACC, rotated vermis, delayed gyration.	ICSA	*TUBA1A*	NM_001270399.1: c.1285G > A	p.(Glu429Lys)	Het	DN	P	PS2, PM1, PM2, PM5, PP3	Yes	Clinical Exome (tier 1)	n.a.	n.a.	Lissencephaly type 3 (#611603; AD)
42	Bowing of‐ and short long bones with unequal aspect of skeleton.	SD	*COL1A2*	NM_000089.3: c.2152G > T	p.(Gly718Cys)	Het	DN	LP	PS2, PM2, PM5, PP3	Yes	In silico disease‐gene panel (tier 1)	n.a.	n.a.	Osteogenesis Imperfecta (Type 2, 3 or 4) (#166210; 259 420; 166 220; AD)
43	Shortening of long bones and small thoracic cage. Possibly a congenital heart defect.	SD	*NIPBL*	NM_133433.3: c.5044C > T	p.(Arg1682*)	Het	DN	P	PVS1, PS2, PM2, PP3	Unclear	Clinical Exome (tier 2)	Postmortem examination: phenotype fitting with Cornelia de Lange syndrome	Yes	Cornelia de Lange syndrome (122 470; AD)
50	Polyhydramnion. Thickened nuchal fold. Fetal hydrops, pleural effusion. Macrosomia. Brachycephaly. ADV, PRUV.	MFCA	*SOS1*	NM_005633.3: c.508A > G	p.(Lys170Glu)	Het	DN	P	PS2, PM1, PM2, PP3, PP5	Yes	Clinical Exome (tier 1)	n.a.	n.a.	Noonan syndrome 4 (#610733; AD)
54	Phocomelia: hands attached to shoulders. Micrognathia. Prenasal thickness. Adduction of lower legs.	SD	*RBM8A*	NM_005105.4: c.‐21G > A Microdeletion 1q21.1	p.(?) haploinsufficient allele	CH	Mat Pat	P P	PS4, PS5, PM3, PP4, PP5	Yes	In silico disease‐gene panel (tier 1)	n.a.	n.a.	Thrombocytopenia absent radius syndrome (TAR) (#274000; AR)

Abbreviations: AD, autosomal dominant; AR, Autosomal recessive; MFCA, multiple fetal congenital anomalies; rWES, rapid whole exome sequencing.

In two additional cases (4%), it was initially unclear if the variant(s) obtained contributed to disease (Figure 1; Table 2; Appendix S1). Follow‐up, using additional clinical information that had become available either through postmortem examination (case #43) or through a next pregnancy of a fetus showing identical ultrasound abnormalities and the same genetic variants (case #16), led to re‐evaluation of variants pathogenicity, and with this, to conclusive diagnoses in both cases.

### 3.5 The influence of rWES in perinatal clinical decision making

**FIGURE 2:**
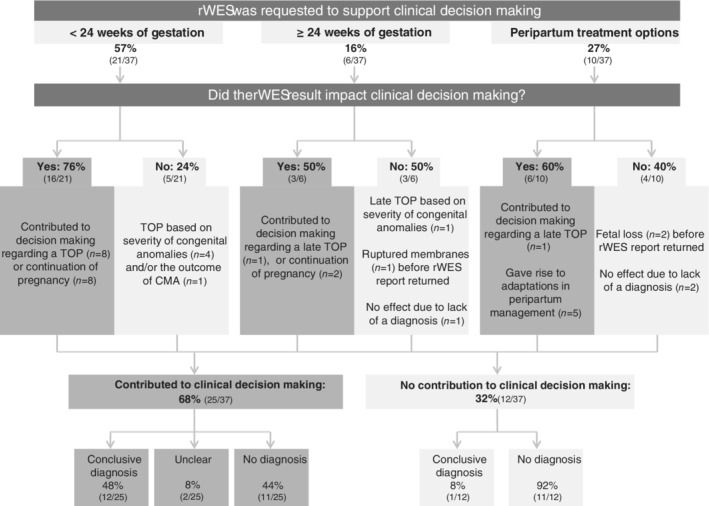
**Impact of rWES on clinical decision making**. Schematic overview of the impact of rWES outcome on clinical decision making. Three main categories for requesting rWES were identified. In each category, rWES impact clinical decision making. From this analysis, it can be clearly shown that rWES impacts clinical decision making, even in the absence of a diagnosis. rWES, rapid whole exome sequencing

A survey to evaluate clinical decision making obtained a response rate of 69% (37/54). Based on diagnostic yield, being 35% for the responders (n = 13/37) and 29% for the non‐responders (n = 5/17) respectively, we concluded this set as the representative for the total cohort (Fisher's Exact *P* = 0.76). For 25 of 37 (68%) responders, it was reported that rWES outcome contributed to clinical decision making, despite the fact that in 44% (11/25) of these cases no genetic cause was identified (Figure 2, Appendix S1).

The main reason to request rWES (n = 21; 57%) was to support clinical decision making before 24 weeks of gestation (Figure 2; Appendix S1). For eight of 21 cases, a negative rWES result provided an argument to continue the pregnancy, whereas in the other 13 cases, pregnancy was terminated. For 9 of 13, this decision was reinforced by the identification of a severe genetic disorder, of which 8 were identified by rWES and the other (#51) by CMA performed in the referring center in parallel to the rWES (#51). In the remaining four cases, TOP was requested following the severity of the identified congenital anomalies.

In 6 of 37 cases (16%), rWES was requested in relation to a late TOP (Figure 2; Appendix S1). In three of them (#2, #15 and #22), rWES results had impact on this decision. For case #15, late TOP was initially rejected, but rWES revealed a severe skeletal dysplasia in the spectrum of Antley‐Bixler syndrome or Pfeiffer syndrome, which led to a review of the initial request. In the other two cases (#2 and 22), the lack of a (severe) genetic diagnosis, however, provided an additional argument for the parents to continue the pregnancy. In the other three cases, rWES results did not impact decision making. In two of them (#22 and #28) no diagnosis was obtained and in the third one (#17), late TOP was already initiated abroad because of the severity of the identified skeletal anomalies prior to the return of the positive rWES results for Kniest dysplasia.

Lastly, in the remaining 10 cases the rWES was requested to guide peripartum rather than prenatal management (Figure 2; Appendix S1). In four cases, the rWES result was unable to have an impact on peripartum management, either because of the absence of a genetic diagnosis (n = 2), or fetal loss (n = 2) before the rWES report returned. Also no genetic cause was identified in the latter two cases. In the other six cases, the outcome of rWES did impact the clinical decision making, albeit for one of these six prenatally rather than peripartum. That is, during the course of the pregnancy of case #12 the prognosis of the identified worsened diaphragmatic hernia and combined with the identified neurodevelopmental disorder (KBG syndrome) it was decided to opt for a TOP. Also cases #1 and #3 presented with a diaphragmatic hernia during fetal ultrasound. Since the rWES for them was negative, there was no reason to withhold invasive treatments. In case #50 the identified genetic disorder (Noonan syndrome type 4) guided adaptation of peripartum management toward more personalized follow‐up diagnostics for identification of additional congenital anomalies related to Noonan syndrome.^25^, ^26^ In case #33, a VUS was identified in the *FRKP* gene, fitting with the clinical suspicion of Walker Warburg syndrome, and guided the decision that not to perform a caesarean section and to withhold life‐sustaining interventions if the child would deteriorate postpartum. In case #7, the rWES report arrived on the first day postpartum after an emergency caesarean section because of fetal distress at 31 weeks of gestation. The severity of the identified genetic disorder (cerebro‐oculo‐facio‐skeletal syndrome type 3, OMIM #616570) was an additional argument toward withholding of life‐sustaining interventions when the child was deteriorating, and this child died on the second day postpartum.

## 4 DISCUSSION

In this study, we aimed to determine the use of rWES in ongoing pregnancies of fetuses with congenital anomalies detected by ultrasound imaging. Using our diagnostic rWES set‐up, we identified the underlying genetic cause in 33% of cases with a median TAT of 10 days. In 68% of the cases, the rWES result contributed to parental and clinical decision making, even when no genetic cause could be identified.

The diagnostic yield of 33% by rWES found in our study is comparable to other studies reporting a diagnostic yield between 18% and 40% when applied to all prenatal abnormalities and without pre‐selecting for certain phenotypes.^17^, ^18^, ^19^, ^20^ However, if such pre‐selecting is performed, even higher diagnostic yields can be obtained, as was shown for fetuses presenting with skeletal anomalies, in whom a diagnosis was reached in 13/16 cases (81%).^14^ Whereas this percentage may seem to be higher than the 55% (11/20) obtained in our subcohort of fetuses with skeletal anomalies, a statistical comparison did not identify difference (Fisher's Exact test, *P* = 0.16). It may however suggest a trend toward a higher diagnostic yield of rWES in fetuses presenting with skeletal anomalies compared to other anomalies. This trend is also observed for the diagnostic interpretation strategy, showing that all conclusive disease‐gene‐panel‐based diagnoses are derived in the subcohort of sketelal dysplasia (11 of 22 cases) when compared with the analysis of the clinical exome (6 of 32 cases; Fishers Exact *P* = 0.020). These observations may however also be the result of our relative small cohort size or clinical representativeness, thereby limiting further firm conclusions from these observations. Similarly, the vast majority of conclusive diagnosis were resulted from point mutations and/or small insertion deletion events, rather than the larger structural variants, which may be explained by a skewed representation of our cohort biased toward cases with skeletal dysplasia.

While the specificity of congenital anomalies already pointed toward the underlying genetic cause in some cases, targeted genetic testing for many of these disorders would have been challenging. An important reason for this is the fact that our current knowledge of genetic disorders is mainly based on postnatal phenotypes of the respective disorders, for which prenatal presentation may differ. This is evident from case #43, presenting with shortening of the long bones and a narrow thorax, a possible heart defect and a mild intra‐uterine growth retardation. This combination of congenital anomalies guided toward the clinical suspicion of a skeletal dysplasia, and therefore the short stature/skeletal dysplasia gene panel was requested. Yet, no (likely) pathogenic causative variant could be identified in these genes. The informed consent allowed for an exome wide analysis which identified a pathogenic variant in the *NIPBL* gene. It was unclear, however, whether the identified shortening of the long bones and possible heart defect could fit with Cornelia de Lange syndrome, although it could explain the intra uterine growth retardation (OMIM #122470). At postmortem examination after a TOP, distinctive recognizable postnatal features, such as hirsutism, upper‐limb reduction defects and craniofacial abnormalities, were identified, supportive in marking the *NIPBL* variant as causative. Whereas similar unanticipated WES diagnoses are a well‐known phenomenon in the postnatal setting, this type of examples in a prenatal setting not only re‐opens the discussion on whether or not to add such genes to a (prenatal) skeletal dysplasia gene panel but also on the clinical use of an exome‐wide strategy, and whether or not the evaluation of variant pathogenicity in a pre‐ and postnatal setting are identical.^27^ Despite these complexities, our results, together with those of others, warrant the adoption of rWES in a prenatal setting, provided that, it is performed in a specialized prenatal (academic) center, with expertise in rWES, and in the presence of a multidisciplinary team consisting of (at least) a clinical laboratory geneticist, a clinical geneticist and a fetal maternal specialist to discuss the rWES results in the clinical context of the fetus' presentation.

One of the most important reasons for the introduction of rWES in a prenatal setting is the possibility to impact prenatal and peripartum clinical decision making. Previously, rWES has already proven to impact clinical decision making for critically ill children, but supportive evidence for prenatal cases is still limited.^10^, ^11^, ^12^, ^13^, ^14^, ^17^, ^28^ Needless to say, prenatal counseling guided by the severity of congenital anomalies alone is often sufficient for parents to opt for TOP, as was also noted for 6 of 37 cases for whom the impact on clinical decision making was determined. In this study, we, however, now also show that rWES outcomes strengthened parental and clinical decision making in 68%, mostly because of the more accurate predictions on the prognosis for parents after the identification of a genetic disorder, and precision medicine for peripartum management. Importantly, impact was obtained for 44% of the cases in whom no causative mutation(s) could be identified. The fact that also a negative rWES has impact on parental and clinical decision making indicates that the efficacy of prenatal rWES should be evaluated by more variables than diagnostic yield alone. This is particularly the case for the clinical subcohort in this study defined as “Others”: in this subcohort no diagnoses were made, which is significantly lower than in the other subcohorts. The latter may suggest that this “Other” cohort does not benefit from rWES. Yet, impact on clinical decision making was imminent in four of six cases for whom the impact was assessed: for the parents of these cases, it was the relief that most monogenic disorders were largely excluded reinforcing their decision to continue the pregnancy.

Recently, two large prospective studies for prenatal rWES in unselected cohorts of fetuses with structural anomalies also showed that rWES can indeed add clinically relevant information to assist current management of a pregnancy, but also highlighted that careful consideration should be given to case selection to maximize clinical usefulness.^29^, ^30^ Based on our experience, the majority of eligible patients were included in our study, however, we did not investigate how often parents did not give consent for rWES. We were able to report trends when comparing different subgroups of fetal congenital anomalies and based on these small numbers we can suggest that performing rWES in skeletal dysplasia, intracerebral structural anomalies and multiple major fetal congenital anomalies are beneficial. This seems in line with the results from Lord et al. en Petrovski et al,^29^, ^30^ and thus that our results contribute to improved patient selection and enhance the clinical utility of rWES for prenatal diagnostics.

## 5 CONCLUSION

We performed rWES in 54 cases of pregnancies in which fetal congenital anomalies by ultrasound imaging were detected, with a median TAT of 10 days. The diagnostic yield in this cohort was 33%. Genetic diagnoses were identified in fetuses who presented with skeletal dysplasia, intracerebral structural anomalies and/or multiple major fetal congenital anomalies. In the majority of cases (68%), the rWES result contributed to clinical decision making, even when no genetic cause could be identified.

## CONFLICT OF INTEREST

The authors report no conflict of interest with this work.

## Supporting information


**APPENDIX** **S1.** Supporting information.

## Data Availability

The data that supports the findings of this study are available in the supplementary material of this article.

## References

[1] Calzolari E , Barisic I , Loane M , et al. Epidemiology of multiple congenital anomalies in Europe: a EUROCAT population‐based registry study. Birth Defects Res A Clin Mol Teratol. 2014;100(4):270‐276. 24723551 10.1002/bdra.23240

[2] Karim JN , Roberts NW , Salomon LJ , Papageorghiou AT . Systematic review of first‐trimester ultrasound screening for detection of fetal structural anomalies and factors that affect screening performance. Ultrasound Obstet Gynecol. 2017;50(4):429‐441. 27546497 10.1002/uog.17246

[3] Skreden M , Skari H , Malt UF , et al. Long‐term parental psychological distress among parents of children with a malformation—a prospective longitudinal study. Am J Med Genet A. 2010;152a(9):2193‐2202. 20803642 10.1002/ajmg.a.33605

[4] Boyle B , Addor MC , Arriola L , et al. Estimating global burden of disease due to congenital anomaly: an analysis of European data. Arch Dis Child Fetal Neonatal Ed. 2018;103(1):F22‐f8. 28667189 10.1136/archdischild-2016-311845 PMC5750368

[5] Wapner RJ , Martin CL , Levy B , et al. Chromosomal microarray vs karyotyping for prenatal diagnosis. N Engl J Med. 2012;367(23):2175‐2184. 23215555 10.1056/NEJMoa1203382 PMC3549418

[6] Callaway JL , Shaffer LG , Chitty LS , Rosenfeld JA , Crolla JA . The clinical utility of microarray technologies applied to prenatal cytogenetics in the presence of a normal conventional karyotype: a review of the literature. Prenat Diagn. 2013;33(12):1119‐1123. 23983223 10.1002/pd.4209 PMC4285999

[7] Neveling K , Feenstra I , Gilissen C , et al. A post‐hoc comparison of the utility of sanger sequencing and exome sequencing for the diagnosis of heterogeneous diseases. Hum Mutat. 2013;34(12):1721‐1726. 24123792 10.1002/humu.22450

[8] Yang Y , Muzny DM , Reid JG , et al. Clinical whole‐exome sequencing for the diagnosis of mendelian disorders. N Engl J Med. 2013;369(16):1502‐1511. 24088041 10.1056/NEJMoa1306555 PMC4211433

[9] Stark Z , Tan TY , Chong B , et al. A prospective evaluation of whole‐exome sequencing as a first‐tier molecular test in infants with suspected monogenic disorders. Genet Med. 2016;18(11):1090‐1096. 26938784 10.1038/gim.2016.1

[10] Willig LK , Petrikin JE , Smith LD , et al. Whole‐genome sequencing for identification of Mendelian disorders in critically ill infants: a retrospective analysis of diagnostic and clinical findings. Lancet Respir Med. 2015;3(5):377‐387. 25937001 10.1016/S2213-2600(15)00139-3 PMC4479194

[11] van Diemen CC , Kerstjens‐Frederikse WS , Bergman KA , et al. Rapid targeted genomics in critically ill newborns. Pediatrics. 2017;140(4):e20162854. 28939701 10.1542/peds.2016-2854

[12] Meng L , Pammi M , Saronwala A , et al. Use of exome sequencing for infants in intensive care units: ascertainment of severe single‐gene disorders and effect on medical management. JAMA Pediatr. 2017;171(12):e173438. 28973083 10.1001/jamapediatrics.2017.3438 PMC6359927

[13] Kingsmore SF , Cakici JA , Clark MM , et al. A randomized, controlled trial of the analytic and diagnostic performance of singleton and trio, rapid genome and exome sequencing in ill infants. Am J Hum Genet. 2019;105(4):719‐733. 31564432 10.1016/j.ajhg.2019.08.009 PMC6817534

[14] Chandler N , Best S , Hayward J , et al. Rapid prenatal diagnosis using targeted exome sequencing: a cohort study to assess feasibility and potential impact on prenatal counseling and pregnancy management. Genet Med. 2018;20:1430‐1437. 29595812 10.1038/gim.2018.30

[15] Rydberg C , Tunon K . Detection of fetal abnormalities by second‐trimester ultrasound screening in a non‐selected population. Acta Obstet Gynecol Scand. 2017;96(2):176‐182. 27714775 10.1111/aogs.13037

[16] Edwards L , Hui L . First and second trimester screening for fetal structural anomalies. Semin Fetal Neonatal Med. 2018;23(2):102‐111. 29233624 10.1016/j.siny.2017.11.005

[17] de Koning MA , Haak MC , Adama van Scheltema PN , et al. From diagnostic yield to clinical impact: a pilot study on the implementation of prenatal exome sequencing in routine care. Genet Med. 2019;21:2303‐2310. 30918357 10.1038/s41436-019-0499-9

[18] Pangalos C , Hagnefelt B , Lilakos K , Konialis C . First applications of a targeted exome sequencing approach in fetuses with ultrasound abnormalities reveals an important fraction of cases with associated gene defects. PeerJ. 2016;4:e1955. 27168972 10.7717/peerj.1955 PMC4860337

[19] Normand EA , Braxton A , Nassef S , et al. Clinical exome sequencing for fetuses with ultrasound abnormalities and a suspected mendelian disorder. Genome Med. 2018;10(1):74. 30266093 10.1186/s13073-018-0582-x PMC6162951

[20] Daum H , Meiner V , Elpeleg O , Harel T . Fetal exome sequencing: yield and limitations in a tertiary referral center. Ultrasound Obstet Gynecol. 2019;53(1):80‐86. 29947050 10.1002/uog.19168

[21] Ali MK , Shazly SA , Ali AH , Abdelbadee AY , Abbas AM . Ultrasonographic soft markers of aneuploidy in second trimester fetuses. Middle East Fertil Soc J. 2012;17(3):145‐151.

[22] Kooper AJ , Faas BH , Feenstra I , de Leeuw N , Smeets DF . Best diagnostic approach for the genetic evaluation of fetuses after intrauterine death in first, second or third trimester: QF‐PCR, karyotyping and/or genome wide SNP array analysis. Mol Cytogenet. 2014;7(1):6. 24428858 10.1186/1755-8166-7-6 PMC3906897

[23] Silva M , de Leeuw N , Mann K , et al. European guidelines for constitutional cytogenomic analysis. Eur J Hum Genet. 2019;27(1):1‐16. 30275486 10.1038/s41431-018-0244-x PMC6303289

[24] Richards S , Aziz N , Bale S , et al. Standards and guidelines for the interpretation of sequence variants: a joint consensus recommendation of the American College of Medical Genetics and Genomics and the Association for Molecular Pathology. Genet Med. 2015;17(5):405‐424. 25741868 10.1038/gim.2015.30 PMC4544753

[25] Lepri F , De Luca A , Stella L , et al. SOS1 mutations in Noonan syndrome: molecular spectrum, structural insights on pathogenic effects, and genotype‐phenotype correlations. Hum Mutat. 2011;32(7):760‐772. 21387466 10.1002/humu.21492 PMC3118925

[26] Romano AA , Allanson JE , Dahlgren J , et al. Noonan syndrome: clinical features, diagnosis, and management guidelines. Pediatrics. 2010;126(4):746‐759. 20876176 10.1542/peds.2009-3207

[27] Diderich K , Joosten M , Govaerts L , et al. Is it feasible to select fetuses for prenatal WES based on the prenatal phenotype? Prenat Diagn. 2019;39(11):1039‐1040. 31506969 10.1002/pd.5522 PMC6899996

[28] Saunders CJ , Miller NA , Soden SE , et al. Rapid whole‐genome sequencing for genetic disease diagnosis in neonatal intensive care units. Sci Transl Med. 2012;4(154):154ra135. 10.1126/scitranslmed.3004041 PMC4283791 23035047

[29] Lord J , McMullan DJ , Eberhardt RY , et al. Prenatal exome sequencing analysis in fetal structural anomalies detected by ultrasonography (PAGE): a cohort study. Lancet. 2019;393(10173):747‐757. 30712880 10.1016/S0140-6736(18)31940-8 PMC6386638

[30] Petrovski S , Aggarwal V , Giordano JL , et al. Whole‐exome sequencing in the evaluation of fetal structural anomalies: a prospective cohort study. Lancet. 2019;393(10173):758‐767. 30712878 10.1016/S0140-6736(18)32042-7

